# Supplementary material for: Molecular Characterization of Plasma HDL, LDL, and VLDL Lipids Cargos from Atherosclerotic Patients with Advanced Carotid Lesions: A Preliminary Report
Source: Int J Mol Sci. 2022 Oct 18;23(20):12449. doi: 10.3390/ijms232012449 (PMC9604033; doi:10.3390/ijms232012449)
Supplement: Supplementary file 1 [file ijms-23-12449-s001.zip › ijms-1907684-supplementary.pdf]

Supplementary Materials

Chemicals and solutions

All reagents and chemicals used in this study were of analytical grade from Merck-Millipore (Darmstadt, Germany), unless otherwise stated.

Ammonium formate (14266), eluent additive for liquid chromatography-mass spectrometry (LC-MS), was purchased from Fluka Analytical (Sigma-Aldrich, St. Louis, MO, USA). LiChrosolv methanol (106035) and propan-2-ol (102781), both hypergrade solvent for LC-MS, were purchased from Supelco (Merck KGaA, Darmstadt, Germany) while chloroform (366927, for HPLC ≥ 99.8%), DMSO (276855) and NaCl (S3014) from Sigma-Aldrich (St. Louis, MO, USA). Milli-Q deionized water was filtered on Millipak filter (0.22 µm, MPGL040001) and purified on a LC-Pak cartridge (C18, LCPAK0001) (all Millipore, Bedford, MA 01730, USA). N,N-dimethylsphingosine (d18:1) (DMS, 860496O) was purchased from Avanti Polar Lipids (Alabaster, USA). The 0.0625 µM DMS solution was prepared by dilution with MeOH/$CHCl_3$ 1/2 of the DMS stock solution (15.3 mM) in DMSO. These solutions were stored and manipulated as previously described [Michelucci E et al., Lipid biomarkers in statin users with coronary artery disease annotated by coronary computed tomography angiography. *Scientific reports* **2021**, 11, (1), 12899].

Table S1. Parameters used for the SRM analysis of the ISTD and 130 lipid species (dwell time 20 msec). CE = cholesteryl ester, Cer = ceramide, DG = diacylglycerol, LPC = lysophosphatidylcholine, LPE = lysophosphatidylethanolamine, PC = phosphatidylcholine, PE = phosphatidylethanolamine, SM = sphingomyelin, TG = triacylglycerol. Q1 = first quadrupole of the mass spectrometer for precursor ion selection. Q3 = third quadrupole of the mass spectrometer for product ion selection. The *m/z* values are calculated using the monoisotopic molecular weights. CEns = collision energies. In product ion type, R = alkyl chain of the fatty acid (of the amide group in the case of Cer).

**Lipid species**		**Precursor ion type**	**Precursor ion *m/z* (Q1)**	**CEn (V)**	**Product ion *m/z* (Q3)**	**Product ion type**
STD	ISTD DMS(d18:1)	$[M+H]^+$	328.3	26	310.3	Precursor$^+$ -H_2O

1	CE(14:0)	$[M+NH_4]^+$	614.6	15	369.4	$Precursor^+$ -RCOOH $-NH_3$
2	CE(14:1)	$[M+NH_4]^+$	612.6	15	369.4	$Precursor^+$ -RCOOH $-NH_3$
3	CE(15:0)	$[M+NH_4]^+$	628.6	15	369.4	$Precursor^+$ -RCOOH $-NH_3$
4	CE(15:1)	$[M+NH_4]^+$	626.6	15	369.4	$Precursor^+$ -RCOOH $-NH_3$
5	CE(16:0)	$[M+NH_4]^+$	642.6	15	369.4	$Precursor^+$ -RCOOH $-NH_3$
6	CE(16:1)	$[M+NH_4]^+$	640.6	15	369.4	$Precursor^+$ -RCOOH $-NH_3$
7	CE(16:2)	$[M+NH_4]^+$	638.6	15	369.4	$Precursor^+$ -RCOOH $-NH_3$
8	CE(17:0)	$[M+NH_4]^+$	656.6	15	369.4	$Precursor^+$ -RCOOH $-NH_3$
9	CE(17:1)	$[M+NH_4]^+$	654.6	15	369.4	$Precursor^+$ -RCOOH $-NH_3$
10	CE(18:0)	$[M+NH_4]^+$	670.6	15	369.4	$Precursor^+$ -RCOOH $-NH_3$
11	CE(18:1)	$[M+NH_4]^+$	668.6	15	369.4	$Precursor^+$ -RCOOH $-NH_3$
12	CE(18:2)	$[M+NH_4]^+$	666.6	15	369.4	$Precursor^+$ -RCOOH $-NH_3$
13	CE(18:3)	$[M+NH_4]^+$	664.6	15	369.4	$Precursor^+$ -RCOOH $-NH_3$
14	CE(20:0)	$[M+NH_4]^+$	698.7	15	369.4	$Precursor^+$ -RCOOH $-NH_3$
15	CE(20:1)	$[M+NH_4]^+$	696.7	15	369.4	$Precursor^+$ -RCOOH $-NH_3$
16	CE(20:2)	$[M+NH_4]^+$	694.6	15	369.4	$Precursor^+$ -RCOOH $-NH_3$
17	CE(20:3)	$[M+NH_4]^+$	692.6	15	369.4	$Precursor^+$ -RCOOH $-NH_3$
18	CE(20:4)	$[M+NH_4]^+$	690.6	15	369.4	$Precursor^+$ -RCOOH $-NH_3$
19	CE(22:0)	$[M+NH_4]^+$	726.7	15	369.4	$Precursor^+$ -RCOOH $-NH_3$
20	CE(22:1)	$[M+NH_4]^+$	724.7	15	369.4	$Precursor^+$ -RCOOH $-NH_3$
21	CE(22:5)	$[M+NH_4]^+$	716.6	15	369.4	$Precursor^+$ -RCOOH $-NH_3$

22	CE(22:6)	$[M+NH_4]^+$	714.6	15	369.4	$Precursor^+$ -RCOOH $-NH_3$
23	Cer(d18:1/16:0)	$[M+H]^+$	538.5	29	264.3	$Precursor^+$ -RCOOH $-H_2O$
24	Cer(d18:1/18:0)	$[M+H]^+$	566.6	29	264.3	$Precursor^+$ -RCOOH $-H_2O$
25	Cer(d18:1/22:0)	$[M+H]^+$	622.6	29	264.3	$Precursor^+$ -RCOOH $-H_2O$
26	Cer(d18:1/23:0)	$[M+H]^+$	636.6	29	264.3	$Precursor^+$ -RCOOH $-H_2O$
27	Cer(d18:1/24:0)	$[M+H]^+$	650.6	29	264.3	$Precursor^+$ -RCOOH $-H_2O$
28	Cer(d18:1/24:1)	$[M+H]^+$	648.6	29	264.3	$Precursor^+$ -RCOOH $-H_2O$
29	DG(30:0)	$[M+NH_4]^+$	558.5	30	523.5	$Precursor^+$ $-H_2O$ $-NH_3$
30	DG(30:1)	$[M+NH_4]^+$	556.5	30	521.5	$Precursor^+$ $-H_2O$ $-NH_3$
31	DG(32:0)	$[M+NH_4]^+$	586.5	30	551.5	$Precursor^+$ $-H_2O$ $-NH_3$
32	DG(32:1)	$[M+NH_4]^+$	584.5	30	549.5	$Precursor^+$ $-H_2O$ $-NH_3$
33	DG(32:2)	$[M+NH_4]^+$	582.5	30	547.5	$Precursor^+$ $-H_2O$ $-NH_3$
34	DG(34:0)	$[M+NH_4]^+$	614.6	30	579.5	$Precursor^+$ $-H_2O$ $-NH_3$
35	DG(34:1)	$[M+NH_4]^+$	612.6	30	577.5	$Precursor^+$ $-H_2O$ $-NH_3$
36	DG(34:2)	$[M+NH_4]^+$	610.5	30	575.5	$Precursor^+$ $-H_2O$ $-NH_3$
37	DG(34:3)	$[M+NH_4]^+$	608.5	30	573.5	$Precursor^+$ $-H_2O$ $-NH_3$
38	DG(36:0)	$[M+NH_4]^+$	642.6	30	607.6	$Precursor^+$ $-H_2O$ $-NH_3$
39	DG(36:1)	$[M+NH_4]^+$	640.6	30	605.6	$Precursor^+$ $-H_2O$ $-NH_3$
40	DG(36:2)	$[M+NH_4]^+$	638.6	30	603.5	$Precursor^+$ $-H_2O$ $-NH_3$
41	DG(36:3)	$[M+NH_4]^+$	636.6	30	601.5	$Precursor^+$ $-H_2O$ $-NH_3$
42	DG(36:4)	$[M+NH_4]^+$	634.5	30	599.5	$Precursor^+$ $-H_2O$ $-NH_3$

43	LPC(16:0)	$[M+H]^+$	496.3	37	184.1	Phosphocholine$^+$
44	LPC(16:0e)	$[M+H]^+$	482.4	37	184.1	Phosphocholine$^+$
45	LPC(16:1)	$[M+H]^+$	494.3	37	184.1	Phosphocholine$^+$
46	LPC(18:0)	$[M+H]^+$	524.4	37	184.1	Phosphocholine$^+$
47	LPC(18:0e)	$[M+H]^+$	510.4	37	184.1	Phosphocholine$^+$
48	LPC(18:1)	$[M+H]^+$	522.4	37	184.1	Phosphocholine$^+$
49	LPC(18:2)	$[M+H]^+$	520.3	37	184.1	Phosphocholine$^+$
50	LPC(20:3)	$[M+H]^+$	546.4	37	184.1	Phosphocholine$^+$
51	LPC(20:4)	$[M+H]^+$	544.3	37	184.1	Phosphocholine$^+$
52	LPC(22:5)	$[M+H]^+$	570.4	37	184.1	Phosphocholine$^+$
53	LPC(22:6)	$[M+H]^+$	568.3	37	184.1	Phosphocholine$^+$
54	LPE(18:0)	$[M+H]^+$	482.3	25	341.3	Precursor$^+$ -phosphoethanolamine
55	PC(32:0)	$[M+H]^+$	734.6	42	184.1	Phosphocholine$^+$
56	PC(32:1)	$[M+H]^+$	732.6	42	184.1	Phosphocholine$^+$
57	PC(32:2)	$[M+H]^+$	730.5	42	184.1	Phosphocholine$^+$
58	PC(34:0)	$[M+H]^+$	762.6	42	184.1	Phosphocholine$^+$
59	PC(34:1)	$[M+H]^+$	760.6	42	184.1	Phosphocholine$^+$
60	PC(34:2)	$[M+H]^+$	758.6	42	184.1	Phosphocholine$^+$
61	PC(34:3)	$[M+H]^+$	756.6	42	184.1	Phosphocholine$^+$
62	PC(36:0)	$[M+H]^+$	790.6	42	184.1	Phosphocholine$^+$
63	PC(36:1)	$[M+H]^+$	788.6	42	184.1	Phosphocholine$^+$

64	PC(36:2)	$[M+H]^+$	786.6	42	184.1	$Phosphocholine^+$
65	PC(36:3)	$[M+H]^+$	784.6	42	184.1	$Phosphocholine^+$
66	PC(36:4)	$[M+H]^+$	782.6	42	184.1	$Phosphocholine^+$
67	PC(36:5)	$[M+H]^+$	780.6	42	184.1	$Phosphocholine^+$
68	PC(38:2)	$[M+H]^+$	814.6	42	184.1	$Phosphocholine^+$
69	PC(38:4)	$[M+H]^+$	810.6	42	184.1	$Phosphocholine^+$
70	PC(38:5)	$[M+H]^+$	808.6	42	184.1	$Phosphocholine^+$
71	PC(38:6)	$[M+H]^+$	806.6	42	184.1	$Phosphocholine^+$
72	PC(40:2)	$[M+H]^+$	842.7	42	184.1	$Phosphocholine^+$
73	PC(40:4)	$[M+H]^+$	838.6	42	184.1	$Phosphocholine^+$
74	PC(40:5)	$[M+H]^+$	836.6	42	184.1	$Phosphocholine^+$
75	PC(40:6)	$[M+H]^+$	834.6	42	184.1	$Phosphocholine^+$
76	PC(40:7)	$[M+H]^+$	832.6	42	184.1	$Phosphocholine^+$
77	PC(40:8)	$[M+H]^+$	830.6	42	184.1	$Phosphocholine^+$
78	PE(34:1)	$[M+H]^+$	718.5	25	577.5	$Precursor^+$ -phosphoethanolamine
79	PE(34:2)	$[M+H]^+$	716.5	25	575.5	$Precursor^+$ -phosphoethanolamine
80	PE(36:1)	$[M+H]^+$	746.6	25	605.6	$Precursor^+$ -phosphoethanolamine
81	PE(36:2)	$[M+H]^+$	744.6	25	603.5	$Precursor^+$ -phosphoethanolamine
82	PE(36:3)	$[M+H]^+$	742.5	25	601.5	$Precursor^+$ -phosphoethanolamine
83	PE(36:4)	$[M+H]^+$	740.5	25	599.5	$Precursor^+$ -phosphoethanolamine
84	PE(38:3)	$[M+H]^+$	770.6	25	629.6	$Precursor^+$ -phosphoethanolamine

85	PE(38:4)	$[M+H]^+$	768.6	25	627.5	$Precursor^+$ -phosphoethanolamine
86	PE(38:5)	$[M+H]^+$	766.5	25	625.5	$Precursor^+$ -phosphoethanolamine
87	PE(38:6)	$[M+H]^+$	764.5	25	623.5	$Precursor^+$ -phosphoethanolamine
88	PE(40:5)	$[M+H]^+$	794.6	25	653.6	$Precursor^+$ -phosphoethanolamine
89	PE(40:6)	$[M+H]^+$	792.6	25	651.5	$Precursor^+$ -phosphoethanolamine
90	SM(32:1)	$[M+H]^+$	675.5	36	184.1	$Phosphocholine^+$
91	SM(32:2)	$[M+H]^+$	673.5	36	184.1	$Phosphocholine^+$
92	SM(34:1)	$[M+H]^+$	703.6	36	184.1	$Phosphocholine^+$
93	SM(34:2)	$[M+H]^+$	701.6	36	184.1	$Phosphocholine^+$
94	SM(35:1)	$[M+H]^+$	717.6	36	184.1	$Phosphocholine^+$
95	SM(36:1)	$[M+H]^+$	731.6	36	184.1	$Phosphocholine^+$
96	SM(36:2)	$[M+H]^+$	729.6	36	184.1	$Phosphocholine^+$
97	SM(36:3)	$[M+H]^+$	727.6	36	184.1	$Phosphocholine^+$
98	SM(37:1)	$[M+H]^+$	745.6	36	184.1	$Phosphocholine^+$
99	SM(38:1)	$[M+H]^+$	759.6	36	184.1	$Phosphocholine^+$
100	SM(38:2)	$[M+H]^+$	757.6	36	184.1	$Phosphocholine^+$
101	SM(38:3)	$[M+H]^+$	755.6	36	184.1	$Phosphocholine^+$
102	SM(40:1)	$[M+H]^+$	787.7	36	184.1	$Phosphocholine^+$
103	SM(40:2)	$[M+H]^+$	785.7	36	184.1	$Phosphocholine^+$
104	SM(41:1)	$[M+H]^+$	801.7	36	184.1	$Phosphocholine^+$
105	SM(41:2)	$[M+H]^+$	799.7	36	184.1	$Phosphocholine^+$

106	SM(41:3)	$[M+H]^+$	797.7	36	184.1	$Phosphocholine^+$
107	SM(42:1)	$[M+H]^+$	815.7	36	184.1	$Phosphocholine^+$
108	SM(42:2)	$[M+H]^+$	813.7	36	184.1	$Phosphocholine^+$
109	SM(42:3)	$[M+H]^+$	811.7	36	184.1	$Phosphocholine^+$
110	SM(42:4)	$[M+H]^+$	809.7	36	184.1	$Phosphocholine^+$
111	SM(43:1)	$[M+H]^+$	829.7	36	184.1	$Phosphocholine^+$
112	SM(43:2)	$[M+H]^+$	827.7	36	184.1	$Phosphocholine^+$
113	SM(43:3)	$[M+H]^+$	825.7	36	184.1	$Phosphocholine^+$
114	TG(48:1)	$[M+NH_4]^+$	822.8	30	549.5	$Precursor^+$ -RCOOH -NH_3 (RCOOH 16:0)
115	TG(48:2)	$[M+NH_4]^+$	820.7	30	549.5	$Precursor^+$ -RCOOH -NH_3 (RCOOH 16:1)
116	TG(50:0)	$[M+NH_4]^+$	852.8	30	579.5	$Precursor^+$ -RCOOH -NH_3 (RCOOH 16:0)
117	TG(50:1)	$[M+NH_4]^+$	850.8	30	577.5	$Precursor^+$ -RCOOH -NH_3 (RCOOH 16:0)
118	TG(50:2)	$[M+NH_4]^+$	848.8	30	549.5	$Precursor^+$ -RCOOH -NH_3 (RCOOH 18:1)
119	TG(50:3)	$[M+NH_4]^+$	846.8	30	575.5	$Precursor^+$ -RCOOH -NH_3 (RCOOH 16:1)
120	TG(50:4)	$[M+NH_4]^+$	844.7	30	547.5	$Precursor^+$ -RCOOH -NH_3 (RCOOH 18:2)
121	TG(52:1)	$[M+NH_4]^+$	878.8	30	579.5	$Precursor^+$ -RCOOH -NH_3 (RCOOH 18:1)
122	TG(52:2)	$[M+NH_4]^+$	876.8	30	577.5	$Precursor^+$ -RCOOH -NH_3 (RCOOH 18:1)
123	TG(52:3)	$[M+NH_4]^+$	874.8	30	575.5	$Precursor^+$ -RCOOH -NH_3 (RCOOH 18:1)
124	TG(52:4)	$[M+NH_4]^+$	872.8	30	575.5	$Precursor^+$ -RCOOH -NH_3 (RCOOH 18:2)
125	TG(52:5)	$[M+NH_4]^+$	870.8	30	573.5	$Precursor^+$ -RCOOH -NH_3 (RCOOH 18:2)
126	TG(54:2)	$[M+NH_4]^+$	904.8	30	605.6	$Precursor^+$ -RCOOH -NH_3 (RCOOH 18:1)

127	TG(54:3)	$[M+NH_4]^+$	902.8	30	603.5	$Precursor^+$ -RCOOH $-NH_3$ (RCOOH 18:1)
128	TG(54:4)	$[M+NH_4]^+$	900.8	30	601.5	$Precursor^+$ -RCOOH $-NH_3$ (RCOOH 18:1)
129	TG(54:5)	$[M+NH_4]^+$	898.8	30	599.5	$Precursor^+$ -RCOOH $-NH_3$ (RCOOH 18:1)
130	TG(54:6)	$[M+NH_4]^+$	896.8	30	599.5	$Precursor^+$ -RCOOH $-NH_3$ (RCOOH 18:2)

Table S2. Means, standard deviations (SD) and percentage of single lipid species within lipid (sub)class evaluated as log2 transformed values.

	**HDL**			**LDL**			**VLDL**		
**Lipidic species**	**Mean**	**SD**	**% species in class**	**Mean**	**SD**	**% species in class**	**Mean**	**SD**	**% species in class**
CE(14:0)	21.01	0.56	4.71	21.80	0.60	4.75	18.74	0.65	4.52
CE(14:1)	17.34	0.74	3.89	18.21	0.84	3.97	16.31	0.93	3.93
CE(15:0)	19.57	0.49	4.39	19.83	0.43	4.32	18.03	0.56	4.35
CE(15:1)	16.58	0.48	3.72	17.12	0.50	3.73	15.87	0.94	3.83
CE(16:0)	25.16	0.33	5.64	25.22	0.41	5.49	23.30	1.04	5.62
CE(16:1)	23.84	0.60	5.35	24.37	0.36	5.31	21.87	0.78	5.27
CE(16:2)	17.84	0.49	4.00	18.62	0.50	4.06	16.41	0.66	3.96
CE(17:0)	19.05	0.44	4.27	19.31	0.38	4.21	17.59	0.39	4.24
CE(17:1)	19.77	0.47	4.43	20.02	0.36	4.36	18.05	0.49	4.35
CE(18:0)	21.59	0.32	4.84	22.05	0.26	4.8	19.69	0.61	4.75
CE(18:1)	25.82	0.30	5.79	25.79	0.45	5.62	23.89	0.76	5.76
CE(18:2)	26.15	0.33	5.86	26.08	0.57	5.68	24.53	0.80	5.91
CE(18:3)	21.48	0.80	4.82	22.47	0.50	4.89	20.20	0.98	4.87
CE(20:0)	22.60	1.96	5.07	23.66	0.62	5.15	19.02	1.07	4.59
CE(20:1)	18.43	1.68	4.13	19.87	0.73	4.33	16.17	1.04	3.9
CE(20:2)	16.85	0.47	3.78	17.36	0.30	3.78	15.92	0.75	3.84
CE(20:3)	20.91	0.76	4.69	21.50	0.36	4.68	19.67	0.86	4.74
CE(20:4)	23.33	1.19	5.23	24.11	0.47	5.25	22.67	0.86	5.47

CE(22:0)	15.64	1.04	3.51	16.41	0.52	3.57	15.62	0.89	3.77
CE(22:1)	18.37	1.59	4.12	19.95	0.56	4.34	16.22	0.97	3.91
CE(22:5)	16.13	1.29	3.62	16.35	0.70	3.56	16.29	0.60	3.93
CE(22:6)	18.52	2.04	4.15	19.06	0.88	4.15	18.75	0.86	4.52
Cer(d18:1/16:0)	15.89	0.74	16.78	16.78	0.52	16.57	16.26	0.62	16.15
Cer(d18:1/18:0)	14.92	0.65	15.76	15.82	0.61	15.61	15.62	0.88	15.51
Cer(d18:1/22:0)	15.66	0.54	16.54	16.85	0.42	16.63	16.76	0.43	16.65
Cer(d18:1/23:0)	15.26	0.52	16.11	16.56	0.36	16.35	16.62	0.43	16.51
Cer(d18:1/24:0)	16.53	0.47	17.45	18.00	0.46	17.77	18.09	0.26	17.96
Cer(d18:1/24:1)	16.44	0.62	17.36	17.29	0.42	17.07	17.35	0.40	17.23
DG(30:0)	14.88	0.69	6.33	14.05	1.30	6.18	16.04	0.96	6.42
DG(30:1)	14.66	0.76	6.24	13.94	1.27	6.14	15.72	1.07	6.29
DG(32:0)	16.78	0.79	7.14	16.03	1.32	7.06	18.19	1.02	7.28
DG(32:1)	16.15	0.54	6.88	15.93	0.83	7.01	17.63	0.68	7.05
DG(32:2)	14.99	0.54	6.38	14.46	1.08	6.37	16.10	0.86	6.44
DG(34:0)	18.12	0.83	7.71	16.93	1.59	7.45	18.97	1.30	7.59
DG(34:1)	18.23	0.41	7.76	18.04	0.37	7.94	19.53	0.37	7.81
DG(34:2)	17.64	0.51	7.51	17.25	0.61	7.6	18.64	0.52	7.46
DG(34:3)	15.09	0.50	6.43	14.70	0.95	6.47	16.15	0.77	6.46
DG(36:0)	18.57	0.80	7.91	17.37	1.53	7.65	19.40	1.58	7.76
DG(36:1)	17.37	0.47	7.39	16.94	0.83	7.46	18.45	0.54	7.38
DG(36:2)	19.28	0.49	8.21	19.02	0.53	8.38	20.05	0.43	8.02
DG(36:3)	17.87	0.54	7.61	17.64	0.46	7.77	18.72	0.49	7.49
DG(36:4)	15.27	0.55	6.5	14.83	0.87	6.53	16.32	0.61	6.53
LPC(16:0)	25.10	0.70	11.11	24.27	0.61	10.91	22.62	0.40	10.99
LPC(16:0e)	18.53	0.80	8.2	17.81	0.63	8.01	16.17	0.67	7.86
LPC(16:1)	19.22	0.96	8.51	18.39	0.56	8.27	16.55	0.92	8.04
LPC(18:0)	24.04	0.55	10.65	23.22	0.65	10.43	21.98	0.36	10.68
LPC(18:0e)	19.72	0.69	8.73	18.94	0.60	8.51	17.60	0.40	8.55
LPC(18:1)	23.02	0.67	10.19	22.12	0.62	9.94	20.69	0.76	10.05
LPC(18:2)	21.47	0.95	9.51	21.30	0.56	9.58	19.65	1.16	9.55
LPC(20:3)	19.42	0.85	8.6	19.48	0.62	8.76	17.98	1.13	8.74
LPC(20:4)	20.67	1.07	9.15	21.39	0.74	9.61	19.06	1.21	9.26
LPC(22:5)	17.03	0.90	7.54	17.56	0.66	7.89	16.25	1.12	7.9
LPC(22:6)	17.62	1.36	7.8	18.00	0.65	8.09	17.26	1.11	8.38
LPE(18:0)	15.79	0.50	100	15.32	0.64	99.99	15.30	1.04	100.01
PC(32:0)	22.88	0.64	4.22	22.93	0.22	4.32	22.47	0.24	4.18
PC(32:1)	23.20	0.70	4.27	22.82	0.58	4.3	22.54	0.64	4.19
PC(32:2)	19.73	0.73	3.63	19.22	0.65	3.62	18.73	0.63	3.48

PC(34:0)	23.77	0.38	4.38	23.38	0.19	4.4	23.38	0.20	4.34
PC(34:1)	27.23	0.40	5.02	26.55	0.39	5	26.89	0.18	5
PC(34:2)	27.27	0.37	5.02	26.53	0.46	4.99	26.91	0.22	5
PC(34:3)	22.51	0.49	4.15	21.85	0.38	4.11	21.69	0.46	4.03
PC(36:0)	22.05	0.37	4.06	21.56	0.29	4.06	21.54	0.32	4
PC(36:1)	25.22	0.38	4.65	24.63	0.28	4.64	24.72	0.34	4.59
PC(36:2)	27.01	0.34	4.98	26.29	0.29	4.95	26.61	0.22	4.95
PC(36:3)	26.55	0.28	4.89	25.91	0.21	4.88	26.18	0.31	4.86
PC(36:4)	26.48	0.57	4.88	26.00	0.33	4.89	26.56	0.38	4.94
PC(36:5)	22.63	1.32	4.17	22.13	0.67	4.17	22.72	0.93	4.22
PC(38:2)	24.52	0.37	4.52	24.62	0.28	4.64	23.79	0.27	4.42
PC(38:4)	26.25	0.55	4.84	25.67	0.46	4.83	26.43	0.41	4.91
PC(38:5)	24.83	0.98	4.57	24.16	0.40	4.55	24.98	0.49	4.64
PC(38:6)	24.68	1.33	4.55	24.46	0.43	4.61	25.48	0.51	4.73
PC(40:2)	19.07	0.49	3.51	18.88	0.44	3.55	17.74	0.40	3.3
PC(40:4)	21.94	0.70	4.04	21.52	0.47	4.05	21.91	0.54	4.07
PC(40:5)	22.75	0.96	4.19	22.31	0.38	4.2	23.24	0.45	4.32
PC(40:6)	23.38	1.30	4.31	22.99	0.51	4.33	24.34	0.57	4.52
PC(40:7)	20.92	1.57	3.85	19.86	0.60	3.74	21.19	0.72	3.94
PC(40:8)	17.93	2.22	3.3	16.87	1.20	3.18	18.15	0.58	3.37
PE(34:1)	16.37	0.44	8.14	16.27	0.53	8.19	17.21	0.42	7.98
PE(34:2)	16.26	0.55	8.09	16.33	0.69	8.23	16.94	0.51	7.86
PE(36:1)	16.13	0.72	8.02	15.54	0.83	7.83	17.30	0.46	8.02
PE(36:2)	18.10	0.64	9	17.56	0.69	8.85	18.91	0.51	8.77
PE(36:3)	16.34	0.77	8.12	16.14	0.82	8.13	16.84	0.56	7.81
PE(36:4)	16.78	0.86	8.34	16.84	0.60	8.49	17.78	0.45	8.25
PE(38:3)	16.69	0.67	8.3	16.36	0.60	8.24	18.04	0.40	8.37
PE(38:4)	18.49	0.86	9.2	18.23	0.64	9.18	20.02	0.51	9.28
PE(38:5)	16.74	1.14	8.33	16.53	0.68	8.33	17.82	0.54	8.27
PE(38:6)	17.00	1.29	**8.46**	17.13	0.68	**8.63**	18.65	0.60	**8.65**
PE(40:5)	15.54	0.78	7.73	15.15	0.89	7.63	17.28	0.52	8.02
PE(40:6)	16.62	1.26	8.27	16.43	0.75	8.27	18.80	0.63	8.72
SM(32:1)	20.98	0.58	**4.02**	21.62	0.50	**4.10**	20.90	0.36	**4.11**
SM(32:2)	17.85	0.58	**3.42**	17.66	0.52	**3.35**	16.95	0.49	**3.33**
SM(34:1)	24.87	0.55	4.76	25.45	0.36	4.83	24.55	0.23	4.83
SM(34:2)	22.20	0.44	4.25	22.59	0.46	4.28	21.83	0.24	4.3
SM(35:1)	19.89	0.55	3.81	20.46	0.47	3.88	19.45	0.28	3.83
SM(36:1)	22.65	0.58	4.34	23.18	0.36	4.4	22.21	0.27	4.37
SM(36:2)	21.89	0.51	4.19	22.18	0.44	4.21	21.42	0.24	4.21
SM(36:3)	18.13	0.42	3.47	17.96	0.46	3.41	17.28	0.33	3.4

SM(37:1)	19.77	0.48	3.79	20.08	0.29	3.81	19.50	0.33	3.84
SM(38:1)	22.61	0.41	4.33	22.88	0.20	4.34	22.17	0.20	4.36
SM(38:2)	21.27	0.43	4.07	21.30	0.27	4.04	20.72	0.30	4.08
SM(38:3)	17.65	0.41	3.38	17.63	0.26	3.34	17.16	0.33	3.38
SM(40:1)	23.96	0.39	4.59	24.39	0.28	4.63	23.36	0.24	4.6
SM(40:2)	23.72	0.39	4.54	23.70	0.22	4.5	22.99	0.19	4.52
SM(41:1)	22.94	0.40	4.39	23.50	0.36	4.46	22.23	0.26	4.37
SM(41:2)	22.92	0.40	4.39	23.04	0.30	4.37	22.12	0.25	4.35
SM(41:3)	21.63	0.40	4.14	21.30	0.27	4.04	21.30	0.33	4.19
SM(42:1)	23.65	0.39	4.53	24.17	0.36	4.58	22.87	0.29	4.5
SM(42:2)	25.71	0.40	4.92	25.90	0.29	4.91	24.93	0.30	4.9
SM(42:3)	24.74	0.35	4.74	24.66	0.30	4.68	23.95	0.25	4.71
SM(42:4)	21.40	0.24	4.1	21.18	0.29	4.02	21.11	0.26	4.15
SM(43:1)	19.91	0.46	3.81	20.50	0.50	3.89	19.06	0.37	3.75
SM(43:2)	21.50	0.49	4.12	21.85	0.42	4.14	20.69	0.33	4.07
SM(43:3)	20.20	0.32	3.87	20.10	0.31	3.81	19.54	0.29	3.85
TG(48:1)	20.13	0.65	5.59	19.66	0.89	5.52	22.57	1.01	5.67
TG(48:2)	19.74	0.79	5.48	18.92	1.32	5.31	21.89	0.90	5.5
TG(50:0)	20.77	0.57	5.77	20.57	0.81	5.78	22.87	0.51	5.74
TG(50:1)	23.15	0.52	6.43	23.06	0.43	6.48	25.16	0.81	6.32
TG(50:2)	22.53	0.53	6.26	21.96	0.69	6.17	24.69	0.79	6.2
TG(50:3)	20.47	0.58	5.69	19.90	0.93	5.59	22.88	0.77	5.75
TG(50:4)	17.52	0.54	4.87	17.29	0.78	4.86	20.08	0.79	5.04
TG(52:1)	22.23	0.44	6.18	22.44	0.28	6.3	24.07	0.70	6.04
TG(52:2)	25.27	0.44	7.02	25.26	0.40	7.09	26.80	0.61	6.73
TG(52:3)	23.45	0.56	6.51	23.22	0.59	6.52	25.70	0.61	6.45
TG(52:4)	20.90	0.72	5.81	20.64	0.72	5.8	23.75	0.66	5.96
TG(52:5)	17.56	0.91	4.88	17.34	0.78	4.87	20.50	0.72	5.15
TG(54:2)	22.27	0.53	6.19	22.84	0.49	6.42	24.15	0.45	6.06
TG(54:3)	24.16	0.62	6.71	24.51	0.58	6.88	25.94	0.49	6.51
TG(54:4)	22.26	0.73	6.18	22.43	0.68	6.3	24.58	0.53	6.17
TG(54:5)	19.43	0.81	5.4	19.01	0.97	5.34	22.21	0.51	5.58
TG(54:6)	18.04	0.65	5.01	16.97	1.41	4.77	20.42	0.55	5.13

Table S3. Results for the differential lipid profile analysis among "hard" (H) and "soft" (S) fractions.

	S.HDL - H.HDL				S.LDL - H.LDL				S.VLDL - H.VLDL			
**Lipidic species**	**logFC**	**AveExpr**	**P.Value**	**adj.P**	**Log_2FC**	**AveExpr**	**P.Value**	**adj.P**	**logFC**	**AveExpr**	**P.Value**	**adj.P**
CE(14:0)	-0.50	21.006	0.020	0.262	-0.27	21.795	0.231	0.567	-0.02	18.744	0.929	0.957
CE(14:1)	-0.41	17.339	0.148	0.371	-0.20	18.207	0.515	0.788	-0.35	16.307	0.308	0.694
CE(15:0)	-0.43	19.565	0.024	0.262	0.07	19.833	0.669	0.844	-0.11	18.027	0.618	0.835
CE(15:1)	-0.32	16.584	0.093	0.311	-0.07	17.123	0.727	0.874	-0.33	15.871	0.344	0.694
CE(16:0)	-0.23	25.161	0.108	0.335	0.18	25.225	0.257	0.567	0.37	23.295	0.341	0.694
CE(16:1)	-0.25	23.839	0.296	0.518	0.07	24.369	0.632	0.839	0.15	21.875	0.607	0.835
CE(16:2)	-0.23	17.843	0.249	0.455	-0.19	18.622	0.317	0.649	-0.02	16.410	0.935	0.957
CE(17:0)	-0.38	19.052	0.030	0.262	0.04	19.310	0.809	0.919	-0.03	17.590	0.825	0.944
CE(17:1)	-0.40	19.771	0.031	0.262	0.13	20.020	0.344	0.649	0.06	18.053	0.756	0.902
CE(18:0)	-0.31	21.593	0.024	0.262	0.03	22.049	0.793	0.919	0.07	19.689	0.774	0.915
CE(18:1)	-0.25	25.819	0.061	0.304	0.29	25.790	0.090	0.364	0.11	23.894	0.690	0.863
CE(18:2)	-0.23	26.153	0.113	0.342	0.26	26.082	0.232	0.567	-0.01	24.532	0.975	0.989
CE(18:3)	-0.02	21.476	0.954	0.972	-0.50	22.469	0.005	0.107	-0.04	20.204	0.911	0.957
CE(20:0)	-1.90	22.603	0.006	0.185	-0.02	23.660	0.944	0.984	-0.42	19.023	0.288	0.694
CE(20:1)	-1.77	18.432	0.003	0.185	-0.21	19.870	0.428	0.716	-0.46	16.174	0.224	0.694
CE(20:2)	-0.08	16.851	0.694	0.827	-0.05	17.362	0.666	0.844	-0.21	15.925	0.458	0.694
CE(20:3)	0.21	20.909	0.479	0.676	-0.07	21.502	0.624	0.837	0.03	19.673	0.934	0.957
CE(20:4)	0.68	23.327	0.125	0.360	-0.21	24.112	0.230	0.567	0.27	22.675	0.407	0.694
CE(22:0)	0.53	15.645	0.173	0.380	-0.29	16.414	0.147	0.478	-0.34	15.620	0.303	0.694
CE(22:1)	-1.53	18.370	0.007	0.185	0.08	19.950	0.712	0.874	-0.30	16.219	0.406	0.694
CE(22:5)	0.86	16.127	0.073	0.304	-0.39	16.351	0.131	0.436	0.03	16.289	0.903	0.957
CE(22:6)	1.32	18.519	0.078	0.304	-0.82	19.058	0.008	0.107	0.25	18.746	0.432	0.694
Cer(d18:1/16:0)	-0.03	15.891	0.918	0.955	0.40	16.782	0.037	0.207	0.52	16.260	0.021	0.347
Cer(d18:1/18:0)	-0.01	14.923	0.974	0.981	0.38	15.816	0.092	0.364	-0.08	15.623	0.815	0.944
Cer(d18:1/22:0)	-0.28	15.661	0.184	0.385	-0.10	16.846	0.534	0.789	-0.04	16.765	0.826	0.944
Cer(d18:1/23:0)	-0.19	15.262	0.372	0.612	-0.17	16.563	0.226	0.567	-0.17	16.623	0.302	0.694
Cer(d18:1/24:0)	-0.19	16.530	0.325	0.556	-0.38	18.004	0.027	0.178	-0.17	18.085	0.114	0.642
Cer(d18:1/24:1)	-0.09	16.442	0.723	0.854	-0.07	17.289	0.661	0.844	-0.03	17.347	0.839	0.949
DG(30:0)	-0.11	14.875	0.689	0.827	0.91	14.046	0.049	0.234	0.12	16.041	0.742	0.893

DG(30:1)	-0.04	14.656	0.882	0.925	1.02	13.936	0.022	0.178	0.19	15.717	0.624	0.835
DG(32:0)	-0.46	16.782	0.127	0.360	0.63	16.029	0.190	0.564	-0.45	18.195	0.230	0.694
DG(32:1)	0.43	16.151	0.039	0.277	0.67	15.926	0.025	0.178	0.45	17.630	0.069	0.599
DG(32:2)	0.27	14.985	0.206	0.397	0.85	14.459	0.027	0.178	0.28	16.101	0.383	0.694
DG(34:0)	-0.60	18.122	0.051	0.304	0.41	16.927	0.484	0.758	-0.75	18.966	0.110	0.642
DG(34:1)	0.23	18.230	0.179	0.385	0.37	18.039	0.007	0.107	0.27	19.529	0.062	0.599
DG(34:2)	0.41	17.638	0.040	0.277	0.42	17.251	0.062	0.288	0.33	18.640	0.091	0.642
DG(34:3)	0.50	15.095	0.009	0.205	0.76	14.699	0.023	0.178	0.22	16.146	0.457	0.694
DG(36:0)	-0.60	18.572	0.049	0.301	0.08	17.374	0.887	0.953	-1.19	19.401	0.034	0.388
DG(36:1)	0.03	17.371	0.879	0.925	0.59	16.941	0.048	0.234	0.04	18.455	0.853	0.956
DG(36:2)	0.16	19.278	0.422	0.645	0.44	19.021	0.024	0.178	0.21	20.050	0.209	0.694
DG(36:3)	0.37	17.874	0.079	0.304	0.20	17.638	0.256	0.567	0.16	18.724	0.392	0.694
DG(36:4)	0.56	15.275	0.007	0.185	0.40	14.828	0.211	0.567	0.05	16.319	0.828	0.944
LPC(16:0)	0.00	25.098	0.986	0.986	0.22	24.267	0.345	0.649	0.32	22.621	0.036	0.388
LPC(16:0e)	-0.20	18.529	0.528	0.711	0.27	17.813	0.253	0.567	0.20	16.175	0.424	0.694
LPC(16:1)	-0.03	19.225	0.933	0.962	0.24	18.393	0.257	0.567	0.55	16.549	0.098	0.642
LPC(18:0)	0.12	24.044	0.597	0.741	0.06	23.215	0.822	0.921	0.16	21.975	0.262	0.694
LPC(18:0e)	-0.04	19.721	0.877	0.925	0.22	18.944	0.333	0.649	0.06	17.602	0.690	0.863
LPC(18:1)	0.16	23.023	0.551	0.723	0.11	22.119	0.645	0.839	0.28	20.690	0.316	0.694
LPC(18:2)	0.02	21.472	0.957	0.972	-0.08	21.304	0.719	0.874	0.21	19.647	0.633	0.835
LPC(20:3)	0.25	19.422	0.442	0.661	0.07	19.484	0.751	0.888	0.40	17.979	0.339	0.694
LPC(20:4)	0.30	20.672	0.458	0.662	0.13	21.389	0.640	0.839	0.42	19.056	0.349	0.694
LPC(22:5)	0.33	17.029	0.344	0.580	-0.01	17.557	0.961	0.984	0.34	16.251	0.405	0.694
LPC(22:6)	1.11	17.621	0.025	0.262	-0.14	18.003	0.563	0.804	0.64	17.256	0.112	0.642
LPE(18:0)	0.13	15.791	0.519	0.711	0.30	15.319	0.214	0.567	-0.39	15.301	0.314	0.694
PC(32:0)	-0.29	22.885	0.242	0.450	-0.04	22.929	0.680	0.850	-0.01	22.471	0.915	0.957
PC(32:1)	-0.11	23.196	0.684	0.827	-0.01	22.822	0.951	0.984	0.18	22.544	0.464	0.694
PC(32:2)	-0.04	19.727	0.877	0.925	-0.28	19.220	0.244	0.567	-0.14	18.731	0.558	0.796
PC(34:0)	-0.21	23.770	0.191	0.395	0.09	23.383	0.335	0.649	0.10	23.383	0.268	0.694
PC(34:1)	-0.21	27.230	0.204	0.397	0.23	26.552	0.129	0.436	0.08	26.888	0.323	0.694
PC(34:2)	-0.21	27.270	0.181	0.385	0.16	26.525	0.376	0.688	-0.09	26.910	0.326	0.694
PC(34:3)	0.15	22.507	0.457	0.662	-0.16	21.847	0.283	0.594	0.08	21.689	0.642	0.835
PC(36:0)	-0.13	22.045	0.411	0.641	0.17	21.561	0.160	0.503	0.14	21.539	0.287	0.694
PC(36:1)	-0.17	25.218	0.299	0.518	0.11	24.627	0.327	0.649	0.13	24.725	0.331	0.694
PC(36:2)	-0.21	27.011	0.162	0.371	0.07	26.288	0.572	0.808	0.00	26.614	0.981	0.989

PC(36:3)	0.04	26.552	0.749	0.875	0.01	25.910	0.954	0.984	0.11	26.178	0.397	0.694
PC(36:4)	0.40	26.480	0.070	0.304	0.09	25.997	0.526	0.789	0.12	26.565	0.415	0.694
PC(36:5)	0.81	22.631	0.097	0.316	-0.19	22.126	0.447	0.725	0.28	22.718	0.423	0.694
PC(38:2)	-0.13	24.523	0.405	0.641	0.14	24.621	0.240	0.567	0.20	23.791	0.075	0.608
PC(38:4)	0.37	26.249	0.091	0.311	0.05	25.667	0.770	0.902	0.15	26.430	0.351	0.694
PC(38:5)	0.69	24.826	0.059	0.304	-0.13	24.162	0.405	0.712	0.24	24.982	0.201	0.694
PC(38:6)	0.87	24.681	0.079	0.304	-0.28	24.461	0.084	0.362	0.23	25.476	0.238	0.694
PC(40:2)	-0.16	19.067	0.412	0.641	0.08	18.881	0.621	0.837	0.21	17.738	0.181	0.694
PC(40:4)	0.38	21.936	0.152	0.371	-0.04	21.523	0.812	0.919	0.17	21.907	0.404	0.694
PC(40:5)	0.66	22.754	0.063	0.304	-0.10	22.306	0.529	0.789	0.22	23.237	0.204	0.694
PC(40:6)	0.88	23.378	0.068	0.304	-0.15	22.986	0.434	0.716	0.23	24.343	0.285	0.694
PC(40:7)	1.27	20.921	0.025	0.262	-0.17	19.858	0.452	0.725	0.37	21.192	0.172	0.694
PC(40:8)	1.34	17.933	0.102	0.324	-0.61	16.874	0.163	0.503	0.22	18.151	0.302	0.694
PE(34:1)	-0.31	16.369	0.085	0.305	-0.23	16.265	0.264	0.571	-0.15	17.213	0.354	0.694
PE(34:2)	-0.32	16.257	0.149	0.371	-0.59	16.329	0.016	0.174	-0.28	16.944	0.139	0.694
PE(36:1)	-0.18	16.134	0.516	0.711	-0.18	15.543	0.557	0.804	-0.39	17.296	0.024	0.347
PE(36:2)	-0.42	18.105	0.086	0.305	-0.59	17.563	0.017	0.174	-0.49	18.911	0.007	0.195
PE(36:3)	-0.17	16.336	0.567	0.730	-0.64	16.145	0.029	0.178	-0.38	16.837	0.069	0.599
PE(36:4)	0.27	16.776	0.414	0.641	-0.58	16.845	0.006	0.107	-0.38	17.784	0.024	0.347
PE(38:3)	0.07	16.690	0.803	0.908	-0.38	16.363	0.081	0.361	-0.23	18.044	0.134	0.694
PE(38:4)	0.19	18.495	0.560	0.728	-0.64	18.232	0.005	0.107	-0.22	20.018	0.262	0.694
PE(38:5)	0.59	16.743	0.166	0.371	-0.56	16.530	0.022	0.178	-0.21	17.821	0.297	0.694
PE(38:6)	0.68	17.005	0.159	0.371	-0.77	17.130	0.001	**0.039**	-0.17	18.651	0.465	0.694
PE(40:5)	0.54	15.539	0.066	0.304	-0.21	15.155	0.515	0.788	-0.20	17.283	0.316	0.694
PE(40:6)	0.66	16.622	0.165	0.371	-0.58	16.425	0.032	0.188	-0.17	18.796	0.461	0.694
SM(32:1)	-0.59	20.981	0.007	0.185	-0.61	21.622	0.000	**0.030**	-0.38	20.898	0.004	0.188
SM(32:2)	-0.51	17.846	0.022	0.262	-0.63	17.658	0.000	**0.030**	-0.50	16.946	0.004	0.188
SM(34:1)	-0.44	24.874	0.037	0.277	-0.28	25.454	0.041	0.213	-0.22	24.552	0.024	0.347
SM(34:2)	-0.33	22.199	0.065	0.304	-0.44	22.589	0.008	0.107	-0.21	21.833	0.034	0.388
SM(35:1)	-0.46	19.888	0.032	0.262	-0.43	20.457	0.012	0.141	-0.29	19.455	0.007	0.195
SM(36:1)	-0.34	22.647	0.133	0.360	-0.18	23.185	0.201	0.567	-0.14	22.206	0.209	0.694
SM(36:2)	-0.26	21.891	0.201	0.397	-0.26	22.179	0.116	0.420	-0.13	21.417	0.208	0.694
SM(36:3)	-0.15	18.129	0.388	0.631	-0.30	17.955	0.088	0.364	-0.08	17.279	0.557	0.796
SM(37:1)	-0.05	19.766	0.786	0.896	-0.31	20.081	0.007	0.107	-0.37	19.496	0.003	0.188
SM(38:1)	-0.33	22.613	0.048	0.301	-0.02	22.881	0.813	0.919	-0.09	22.166	0.288	0.694

SM(38:2)	-0.26	21.269	0.138	0.360	-0.09	21.297	0.429	0.716	-0.12	20.715	0.328	0.694
SM(38:3)	-0.04	17.653	0.811	0.909	-0.08	17.633	0.480	0.758	-0.06	17.164	0.653	0.841
SM(40:1)	-0.28	23.963	0.087	0.305	0.02	24.392	0.856	0.940	-0.01	23.357	0.933	0.957
SM(40:2)	-0.25	23.720	0.127	0.360	0.02	23.703	0.863	0.940	0.03	22.990	0.738	0.893
SM(41:1)	-0.35	22.938	0.032	0.262	-0.12	23.497	0.400	0.712	-0.09	22.234	0.432	0.694
SM(41:2)	-0.25	22.916	0.136	0.360	-0.06	23.040	0.620	0.837	-0.01	22.120	0.921	0.957
SM(41:3)	0.12	21.629	0.478	0.676	-0.11	21.296	0.344	0.649	0.01	21.299	0.925	0.957
SM(42:1)	-0.23	23.652	0.157	0.371	0.01	24.174	0.949	0.984	0.05	22.870	0.685	0.863
SM(42:2)	-0.09	25.706	0.590	0.741	0.14	25.902	0.239	0.567	0.20	24.928	0.089	0.642
SM(42:3)	-0.03	24.743	0.848	0.925	0.07	24.658	0.555	0.804	0.14	23.954	0.171	0.694
SM(42:4)	0.15	21.399	0.198	0.397	-0.01	21.181	0.956	0.984	0.04	21.114	0.731	0.893
SM(43:1)	-0.34	19.910	0.069	0.304	-0.38	20.497	0.040	0.213	-0.13	19.057	0.381	0.694
SM(43:2)	-0.29	21.505	0.138	0.360	-0.18	21.849	0.283	0.594	-0.06	20.691	0.643	0.835
SM(43:3)	0.02	20.202	0.875	0.925	-0.11	20.104	0.396	0.712	0.06	19.544	0.614	0.835
TG(48:1)	-0.13	20.129	0.599	0.741	0.39	19.662	0.227	0.567	0.23	22.569	0.546	0.796
TG(48:2)	-0.10	19.740	0.754	0.875	0.75	18.920	0.115	0.420	0.00	21.885	0.994	0.994
TG(50:0)	-0.21	20.773	0.348	0.580	0.24	20.571	0.429	0.716	-0.07	22.872	0.731	0.893
TG(50:1)	-0.24	23.148	0.237	0.447	0.08	23.061	0.622	0.837	0.21	25.157	0.486	0.718
TG(50:2)	0.04	22.533	0.854	0.925	0.40	21.956	0.112	0.420	0.35	24.686	0.236	0.694
TG(50:3)	0.15	20.466	0.526	0.711	0.52	19.897	0.120	0.421	0.29	22.881	0.315	0.694
TG(50:4)	0.23	17.520	0.286	0.510	-0.01	17.293	0.985	0.987	0.04	20.084	0.886	0.957
TG(52:1)	-0.11	22.229	0.538	0.714	0.09	22.438	0.435	0.716	0.26	24.075	0.325	0.694
TG(52:2)	-0.12	25.271	0.530	0.711	0.20	25.256	0.191	0.564	0.21	26.803	0.368	0.694
TG(52:3)	0.12	23.447	0.587	0.741	0.21	23.222	0.351	0.652	0.26	25.697	0.258	0.694
TG(52:4)	0.22	20.903	0.428	0.646	0.00	20.641	0.987	0.987	0.18	23.751	0.463	0.694
TG(52:5)	0.43	17.561	0.208	0.397	-0.15	17.339	0.612	0.837	0.21	20.499	0.447	0.694
TG(54:2)	-0.06	22.274	0.764	0.879	0.03	22.839	0.868	0.940	0.08	24.154	0.634	0.835
TG(54:3)	-0.04	24.165	0.863	0.925	0.08	24.508	0.721	0.874	0.11	25.940	0.563	0.796
TG(54:4)	0.22	22.259	0.448	0.661	-0.09	22.431	0.733	0.874	0.16	24.584	0.433	0.694
TG(54:5)	0.35	19.432	0.255	0.461	-0.01	19.009	0.983	0.987	-0.03	22.207	0.895	0.957
TG(54:6)	-0.12	18.039	0.637	0.781	0.09	16.971	0.854	0.940	-0.33	20.421	0.104	0.642

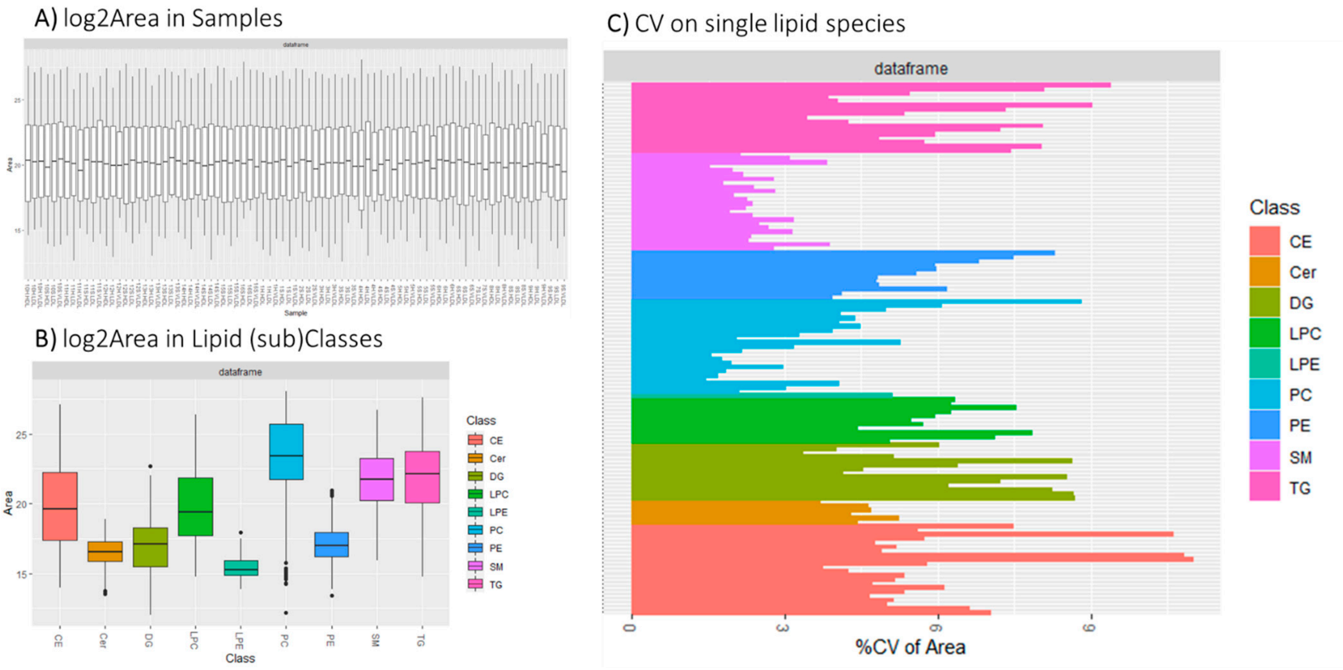

Figure S1. Quality control assessments on lipidomics experimental data. Total lipid content in samples after normalization (A), distribution for each lipid (sub)class (B), and variation coefficients (CV%) for single lipid species (C).

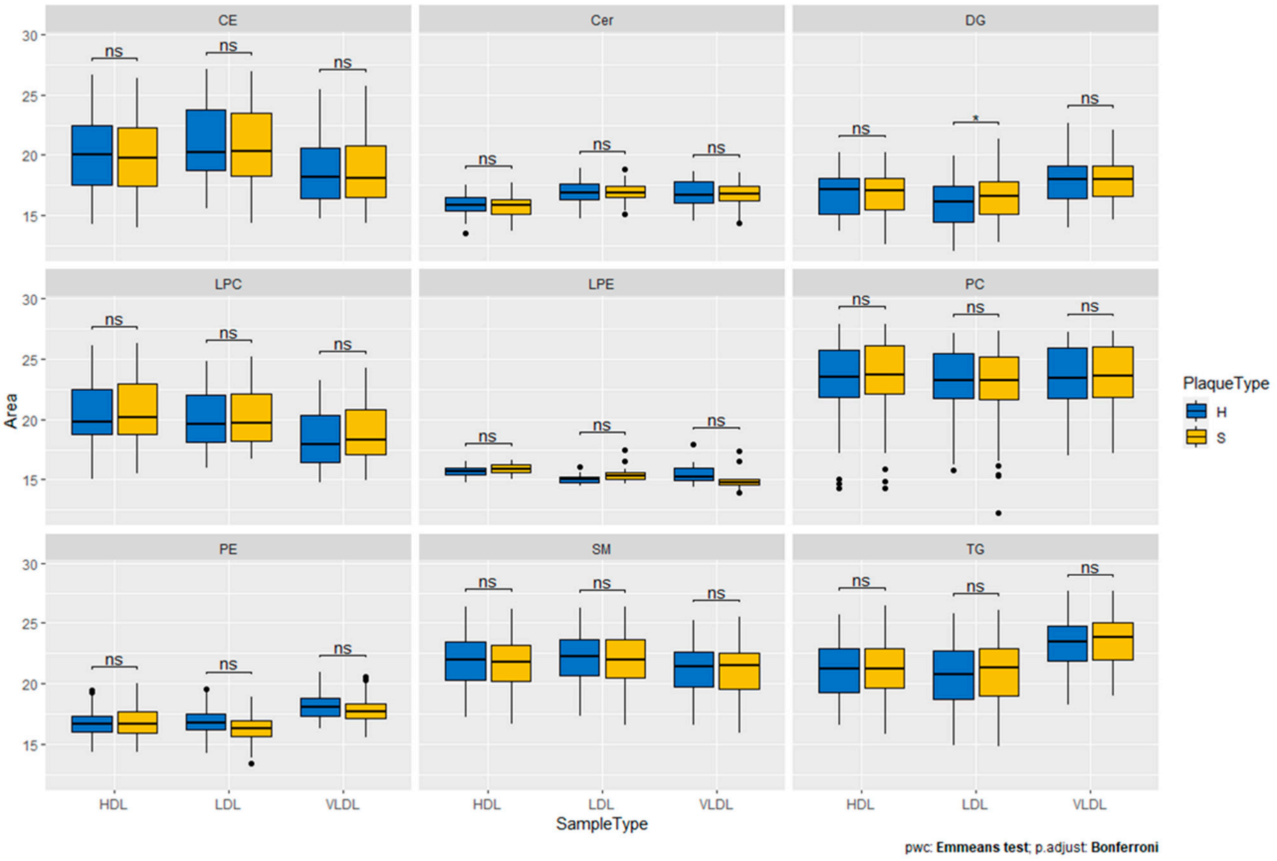

Figure S2. Distribution of the nine different lipid classes in each lipoprotein fraction following sorting for plaque typology. * p-value < 0.05.

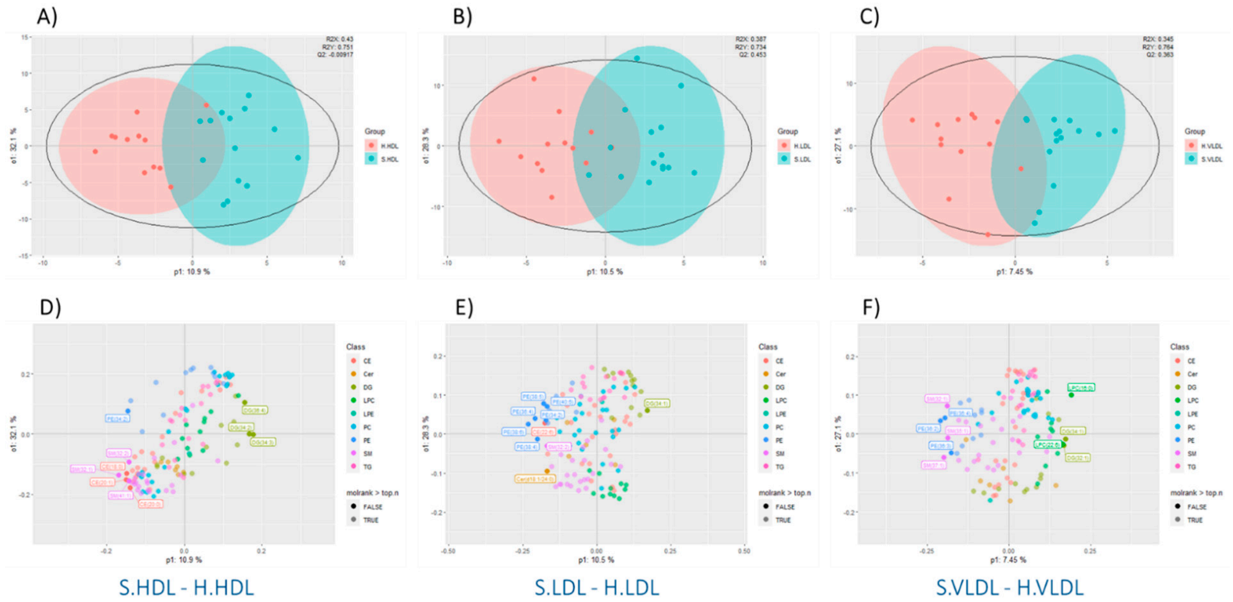

Figure S3. Orthogonal partial least-squares discriminant analysis (OPLS-DA) for HDL, LDL, and VLDL fractions according to the plaque typology. (A, B, C) OPLS-DA plot for each lipoprotein fraction type (HDL, LDL, VLDL respectively) using the plaque typology as a grouping variable (S., "soft" and H., "hard"). The ellipse around each group shows the group's 95% confidence region. (D, E, F) Loading plots highlight the top 10 lipids contributing to the separation between different groups.
